# Long-Term Performance of Concrete Made with Different Types of Cement under Severe Sulfate Exposure

**DOI:** 10.3390/ma16010240

**Published:** 2022-12-27

**Authors:** Ahmed M. Tahwia, Rowyda M. Fouda, Mohamed Abd Elrahman, Osama Youssf

**Affiliations:** Structural Engineering Department, Faculty of Engineering, Mansoura University, Mansoura 35516, Egypt

**Keywords:** durability of concrete, sulfate exposure, blast furnace cement, sulfate resisting Portland cement, Portland cement, silica fume

## Abstract

Concrete sulfate attack is of great interest as it represents one of the main reasons of concrete deterioration and poor durability for concrete structures. In this research, the effect of different cement types on concrete sulfate resistance was investigated. This included three concrete classes, namely, low strength concrete, medium strength concrete, and high strength concrete. Blast furnace cement (BFC), sulfate resisting Portland cement (CEM I-SR5), and ordinary Portland cement (OPC) were used in a total of eighteen concrete mixes. Three binder contents of 250 kg/m^3^, 350 kg/m^3^, and 450 kg/m^3^ and a constant silica fume (SF) content were applied in this experimental study. The water/binder (w/b) ratio was varied between 0.4 and 0.8. Concrete specimens were immersed in highly severe effective sodium sulfate solutions (10,000 ppm) for 180 days after standard curing for 28 days. The fresh concrete performance was evaluated through a slump test to attain proper workability. Concrete compressive strength and mass change at 28 days and 180 days were measured before and after immersion in the solution to evaluate the long-term effect of sulfate attack on the proposed concrete durability. Scanning electron microscopy (SEM) analysis was conducted to study the concrete microstructure and its deterioration stages. The obtained results revealed that BFC cement has the best resistance to aggressive sulfate attacks. The strength deterioration of BFC cement was 3.5% with w/b of 0.4 and it increased to about 7.8% when increasing the w/b ratio to 0.6, which are comparable to other types of cement used. The findings of this research confirmed that the quality of concrete, specifically its composition of low permeability, is the best and recommended protection against sulfate attack.

## 1. Introduction

Concrete is one of the most usable and durable cement-based materials in the construction field with different applications [1,2,3,4,5,6,7]. It has several advantages over other construction materials such as high strength, fire resistance, and high durability which extends the service life of structures. Numerous researchers have recently focused on concrete durability and factors affecting it, and on finding different methods to improve durability-related characteristics. Concrete durability can be evaluated by its performance under severe weather conditions, abrasion, chemical and physical attacks, and other deterioration processes [8,9]. Improving the durability of concrete increases its serviceability and keeps its original characteristics even under severe environmental attacks. According to ACI 201-2R-2008 [9], concrete is mostly exposed to both physical and chemical sulfate attacks that accelerate the deteriorating process in weak concrete. Amongst the most essential matters concerning the durability of concrete structures are their resistances to sulfate exposure. The cement matrix suffers from deterioration due to spalling, softening, and expansion in the sulfate environment [10,11,12]. There are four main factors that affect concrete resistance to sulfate attack; (1) Cement type; (2) Sulfate type and concentration; (3) Quality of concrete; (4) Exposure conditions [12]. This attack occurs when concrete is exposed to a sulfate source through rainwater, soil, or groundwater. The attack of sulfate is typically manifested through cracking of concrete and spalling, followed by expansions and strength reduction. Several factors, including water to cement (w/c) ratio, transportation characteristics, mix components, and cement fineness and composition govern the performance of concrete when exposed to aggressive sulfate attack [12]. Some types of soil with concentrations of sulfate might be categorized as moderate or severe conforms to ACI 201-2R-2008 [9], as shown in Table 1, which can have adverse effects on concrete service life. Camacho and Afif [13] indicated sulfate proportions in soils as 0.11% to 0.70% and in groundwater and wastewaters as 288 to 10,000 parts per million [14]. Calcium, potassium, sodium, and magnesium ions were observed in association with the other ions that originated in seawater, soil, and groundwater [15]. Hazardous ions such as sulfate ions enter concrete and form ettringite having a higher volume than the initial components in the reaction, which began due to the reaction with calcium hydroxide (Ca(OH)_2_) and tri-calcium aluminate hydrate (C_3_AH_6_) [15]. The ettringite and gypsum generated due to a sulfate attack have much more volume (1.2–2.2 times) than the original chemical reactions. This can cause cracking and softening in concrete structures and ultimately decrease their durability [15]. All of the above responses are followed by a significant decrease in the strength of concrete.

Calcium hydroxide (Ca(OH)_2_) reacts with sulfate ions (SO4^2−^) in the pore solution, producing gypsum in the case of sodium sulfate (Na_2_SO_4_) attack, leading to strength deterioration of concrete. As a result of this reaction, ettringite is created as a secondary product that increases volume, resulting in expansion and cracking owing to the reaction with tricalcium aluminate (C_3_A), monosulfate (C_4_A¯SH_12_), and calcium aluminate hydrate (C_4_AH_13_) [16]. However, Ca(OH)_2_ reacts with magnesium sulfate in case of magnesium sulfate (MgSO_4_) attack to create brucite (Mg(OH)_2_) and gypsum. Calcium silicate hydrate (C-S-H) and ettringite are thus destabilized at low pH levels of brucite. Therefore, the reaction of C-S-H with MgSO_4_ is accelerated to produce MgSO_4_ and acts rapidly with C-S-H to silica gel (S_2_H) and gypsum. Additional amounts of free lime have existed in the system, which can respond to react with MgSO_4_ to produce more brucite; consequently, a higher content of brucite and gypsum is created in the microstructure of the concrete matrix. With such a high increase in brucite concentration, it reacts with S_2_H. As a result, C-S-H gradually decomposes, forfeits its lime, and transforms to another phase, non-cementitious M-S-H (magnesium silicate hydrate) [17]. To deal with sulfate attacks in many countries, blast furnace cement (BFC) improves sulfate and chloride resistance due to its stable characteristics under such severe conditions. Frearson [18] reported that ordinary Portland cement (OPC) suffers tremendous damage due to an aggressive attack of sulfate. He studied the effect of using different water to binder (w/b) ratios and different contents of granulated-blast furnace slag (GGBFS) as substitutes of OPC on concrete resistance to the attack of sulfate. The result of the content of slag was concluded to be greater than the effect of the w/b ratio on improving resistance to sulfate [19]. In other research work, the high content of slag in concrete has been reported to increase resistance to sulfates by reducing the permeability and densifying the microstructure, and refining the pores, which reduce the penetration of water and severe ions into concrete. Improved concrete resistance to sulfate and chloride attacks associated with the addition of optimum slag content is primarily owing to the decrease in tricalcium aluminate and rising in concentration of slag in cement-concrete. Furthermore, incorporation of particles of slag in the cement-based material system reduced the content of calcium hydroxide and increased the concentration of C-S-H phases, which improves concrete mechanical characteristics and reduces its reaction rate between sulfate ions and calcium hydroxide [19]. Blended types of cement composed of OPC mixed with various mineral additives such as slag, fly ash, or natural pozzolana showed good resistance to severe sulfate attack due to pozzolanic reaction, densifying the matrix, reducing C_3_A content, and minimizing CH content in the system [20,21]. Although both fly ash and blast furnace slag achieved an enhancement in concrete sulfate resistance, the contribution varied with the initial moist curing time [22]. In addition, the incorporation of silica fume (SF) to concrete enhances the durability aspects of concrete through refining the pore system [22], decreasing the permeation, minimizing harmful substances diffusion, and consuming calcium hydroxide leading to better resistance to severe sulfate exposure. These parameters help protect the reinforcement embedded in concrete from severe corrosion [23]. It is also reported that the addition of 5–10% of SF to replace cement by mass can help significantly to improve resistance to the attack of sodium sulfates without any indication to concrete spalling even after 365 days in solution with 5% concentration of sodium sulfate [24,25]. Replacing cement with fly ash causes concrete mass loss that is owing to the less formation of the components like Ca (OH)_2_ during the process of deterioration [25].

Recently, several studies have been carried out to reduce the harmful impact of sulfate attack on concrete using supplementary cementitious materials as well as nanomaterials [26,27,28,29,30,31]. Most of the researchers focused on three ways to reduce concrete deterioration under sulfate attack. The first one was by minimizing the portlandite content in the pore solution. The second way was by decreasing C_3_A content, and the third one was by reducing the permeability of concrete to prevent the sulfate solution from penetrating the concrete [26]. Incorporation of fly ash, metakaolin, silica fume, and slag can mitigate sulfate attack significantly depending on the replacement level and sulfate attack degree [26,27,28]. However, there are contrary effects on concrete durability such as reducing alkalinity of the pore solution. On the other hand, it was reported that incorporation of nanosilica and nano metakaolin reduces the strength loss and improves the resistance of concrete to sulfate attack [29,30]. Baldeman et al. [31] studied the influence of limestone on sulfate resistance and concluded that the performance of concrete can be improved when the replacement level is lower than 50%. The performance of cement-based materials under sulfate attack is dependent mainly on the mix composition as well as the exposing degree and period.

## 2. Research Scope

Various publications discussed the performance of different types of concrete under sulfate attack; however, according to the authors’ best knowledge, there is no investigations comparing sulfate resistance of different types of cement and different concrete qualities. This research investigates the influence of concrete quality and three different types of cement on sulfate resistance of concrete with various strength levels. Three strength classes of concrete; low strength, medium strength, and high strength have been prepared and tested. In addition, the impact of SF and w/b ratio on concrete resistance to sulfate has been studied intensely and compared with other concrete components. Prepared mixes were subjected to very severe Na_2_SO_4_ solutions (10,000 ppm) for 180 days after 28 days of water curing. A Slump test was performed in order to control the workability of the produced concrete. Compressive strength of concrete mixes was determined at 28 days as an indicator for mechanical properties. Durability was assessed using compressive strength loss and mass change at 180 days. Scanning electron microscopy (SEM) was implemented to analyze the concrete microstructure and the formed phases due to severe sulfate attacks. The SEM imaging was carried out using a JSM 6510 lv microscope (made by JEOL Ltd., Tokyo, Japan) at an acceleration voltage of 30 kV.

## 3. Experimental Study

### 3.1. Raw Materials and Mix Design

#### 3.1.1. Binder

**Figure 1:**
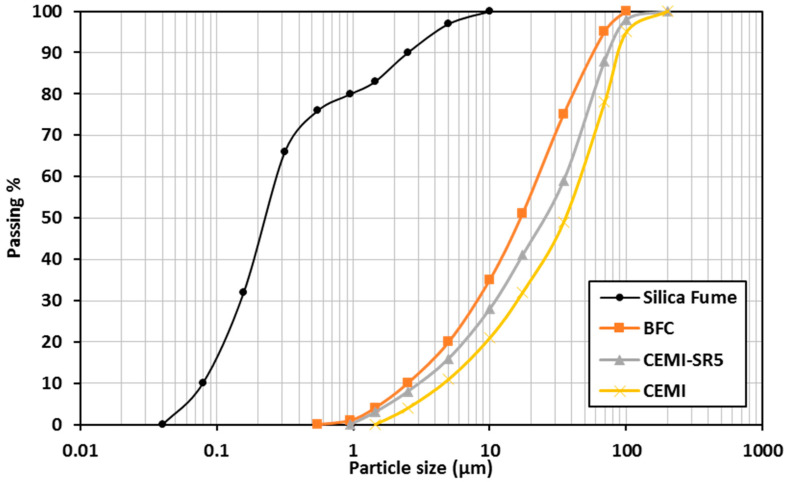
Particle size distribution of the binder used.

In this investigation, the performance of three different types of cement were examined under sever sulfate attack. Ordinary Portland cement grade 42.5 conforming to EN 197-1 (2011) [32] was used as a reference cement. The second type was sulfate resisting Portland cement (CEMI 42.5N–SR5) which is indicated for usage in concrete subjected to high sulfate levels and conformed to EN 197-1 (2011) [32]. The third type was blast furnace cement (BFC) type CEM III/A grade 42.5 which consists of 50% ground granulated-blast furnace slag (GGBFS) by mass and conformed to EN 197-1 (2011) [32]. Table 2 shows the characteristics of the cements used. In this investigation, locally available SF from “EFACO Egypt” that consists of 98% silicon dioxide (SiO_2_) was used. The fineness, bulk density, and specific gravity of SF were 23,420 m^2^/kg, 8.0 t/m^3^, and 2.2, respectively. Figure 1 shows the particle size distribution of all binder materials used in this study.

#### 3.1.2. Aggregates

**Figure 2:**
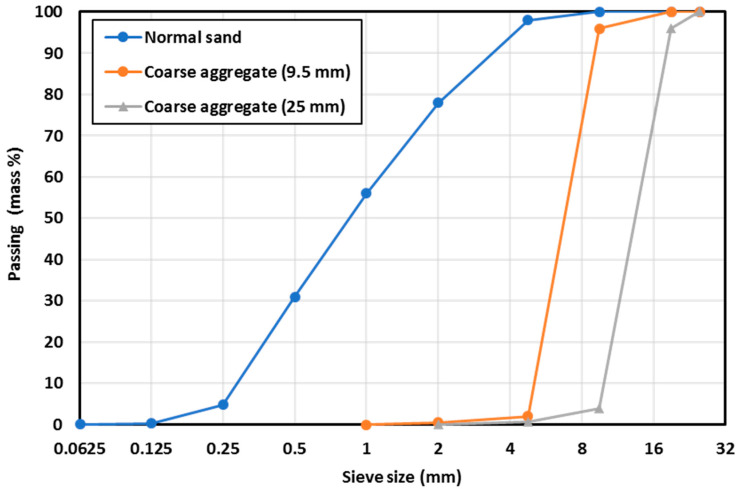
Grading curves for all aggregates used.

In this research, crushed dolomite stone was utilized as coarse aggregate with two nominal maximum sizes of 9.5 mm and 25 mm. In addition, natural siliceous sand was used as fine aggregate with a maximum particle size of 2.36 mm and fineness modulus of 2.57. Table 3 summarizes the physical characteristics of the fine and coarse aggregates. Figure 2 shows the grading curves of the aggregates used as measured in the laboratory.

### 3.2. Mixes Proportioning

Eighteen different concrete mixes were designed, prepared, and tested. Concrete mixes with three strength classes were mixed and examined after sulfate attack. The binder contents were 250 kg/m^3^, 350 kg/m^3^, and 450 kg/m^3^ for low strength concrete, medium strength concrete, and high strength concrete, respectively. SF content was constant at 10% by weight of binder in all mixes. The w/b ratio was varied between 0.4 and 0.8 depending on the strength class and the existence of high range water reducer (HRWR). The HRWR was used in some mixes with dosage of 1% by weight of cement in order to achieve proper workability. In those mixes, the w/b ratio decreased to 0.40–0.65. For low strength concrete, local crushed dolomite was used as coarse aggregate with a nominal maximum size of 25 mm. For medium and high strength concrete mixes, local crushed dolomite was used as coarse aggregate with a nominal maximum size of 9.5 mm. Normal sand with fineness modulus of 2.57 was used as fine aggregate. The water absorption of coarse aggregate was 0.5 % while it was 1.1% for fine aggregate. Additional amount of water equaling to aggregate water absorption has been added to the mixing water in order to prevent negative effects on concrete workability. Nine mixes were prepared and three mixes were considered as control mixes (with superplasticizer) with w/b ratio of 0.65 for low strength concrete, 0.45 for medium strength concrete, and 0.4 for high strength concrete. The other nine mixes had w/b of 0.80 for low strength concrete, and 0.60 for both medium and high strength concrete mixes. Slump test was utilized to measure the workability of fresh concrete. Cubical specimens of 100 mm³ were prepared from each mix and water cured for 28 days, then some of the cube specimens were kept in very severe sodium sulfate solutions (10,000 ppm) for 180 days according to ACI 318-19 [33]. Compressive strength of concrete mixes was determined at 28 days. Durability was assessed using compressive strength loss and mass change at 180 days. SEM analysis was implemented to analyze concrete microstructure and the formed phases due to severe sulfate attacks. Details of various mix proportions and their codes are displayed in Table 4.

### 3.3. Testing of Fresh Concrete

The performance of the fresh concrete was evaluated through a slump test to attain proper workability according to ASTM C-143 [34]. This test was performed directly after concrete mixing to ensure that all mixes achieved the targeted workability of ˃50 mm.

### 3.4. Testing of Hardened Concrete

#### 3.4.1. Compressive Strength Test

In this investigation, a compressive strength test was carried out at 28 days and 180 days conforming to BS EN 12390-3-19 [35]. In addition, strength deterioration due to severe sulfate exposure is determined by measuring the compressive strength of concrete after 180 days of soaking in sulfate solutions. The tests were performed on concrete 100 mm³ cubes. All concrete specimens were cured for 28 days in water with controlled temperature of 21 ± 2 °C. Three specimens of each mix were tested, and the mean value was considered in this investigation. All specimens have been tested in a compressive hydraulic test machine.

#### 3.4.2. Mass Change Test

The change in concrete mass can be considered a good sign to assess the concrete resistance to sulfate attack. Mass loss expresses the deterioration degree of hydration products. It can be viewed as an indicator of the degree of spalling of the concrete sample during severe sulfate exposure. Mass change can be determined by measuring specimen mass, which was determined utilizing a digital scale with high precision of 0.01 g. Prior to starting their measurement, concrete specimens were dried entirely to constant mass. The following formula (Equation (1)) was used to calculate the mass change in concrete specimens [36]:

(1) $$W=\frac{M_{t}-M_{0}}{M_{0}}\times 100$$

where *W* is the change in specimen mass, *M*_0_ is the initial mass of the concrete specimen before immersion, and *M_t_* is the specimen mass after immersion in a sulfate solution. In this study, the change in the specimen’s masses was determined at 28 days before the sulfate exposure as control value, and at 56, 90, and 180 days after the sulfate exposure.

#### 3.4.3. Scanning Electron Microscopy

The microstructure characteristics of concrete are the main parameters governing the performance of different types of concrete under such aggressive conditions. Therefore, in this study, microstructural characterization has been performed using SEM analysis. It has been used to analyze transition zone characteristics, the phase’s formation and deterioration, and crack propagation. In this investigation, SEM measurements were performed in the laboratory of Mansoura University, Egypt using a device named “JEOL JSM-651OLV, OXFORD” at magnifications of 5000 and 6500. The measurement focused on studying the influence of sulfate attacks on concrete characteristics. The test has been conducted at 180 days to study the characteristics of concrete microstructure before and after the sulfate attack.

## 4. Results and Discussion

### 4.1. Workability

**Figure 3:**
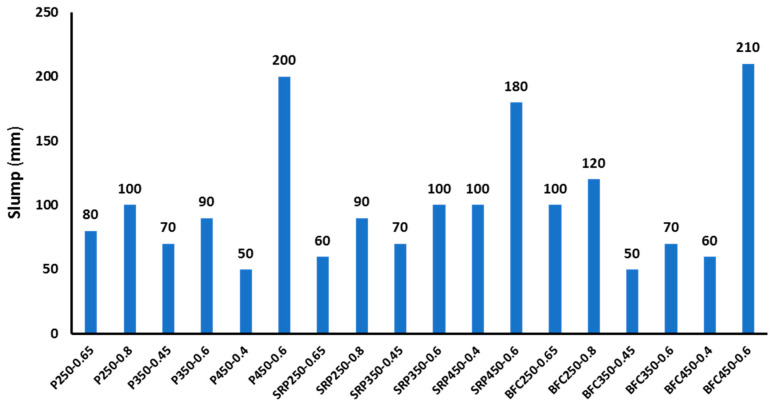
Experimental results of the slump test for all mixes.

Figure 3 shows the experimental results of the slump test for different concrete mixes. It is clear from the figure that all mixes achieved the targeted slump > 50 mm. The main parameter governing the workability of fresh concrete is the w/b ratio. At the same HRWA contents, the slump values directly proportioned with the w/b ratio. For concrete mixes with strength level of 250 kg/cm^2^, at the same w/b ratio and HRWA content, mixes prepared with BFC cement exhibited the highest slump values compared to other types of cement. The same trend can be detected for mixes with strength classes of 350 kg/cm^2^ and 450 kg/cm^2^. It is obvious from the results that the role of cement type and characteristics on fresh concrete workability is marginal while the w/b ratio and HRWA content are the main parameters with strong impact on concrete slump.

### 4.2. Compressive Strength

**Figure 4:**
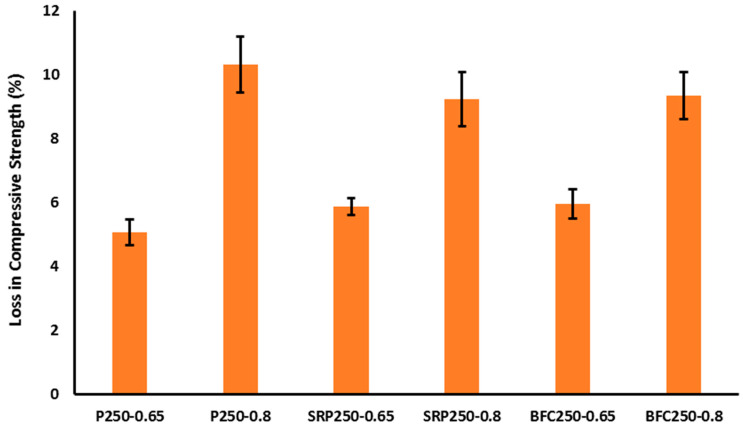
Strength loss of “low strength concrete class” after sulfate exposure.

**Figure 5:**
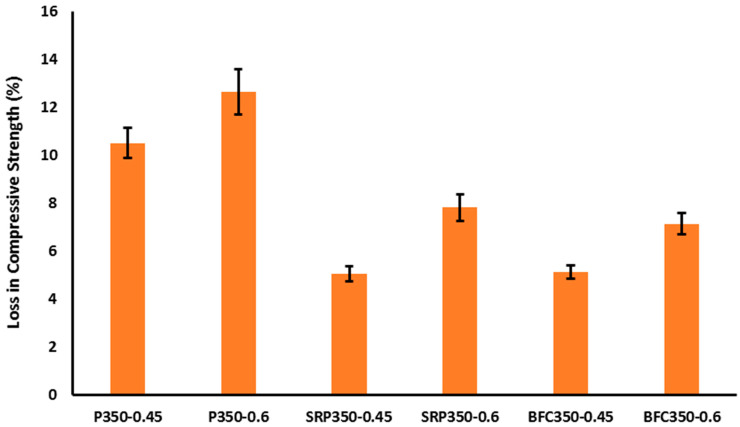
Strength loss of “medium strength concrete” class after sulfate exposure.

**Figure 6:**
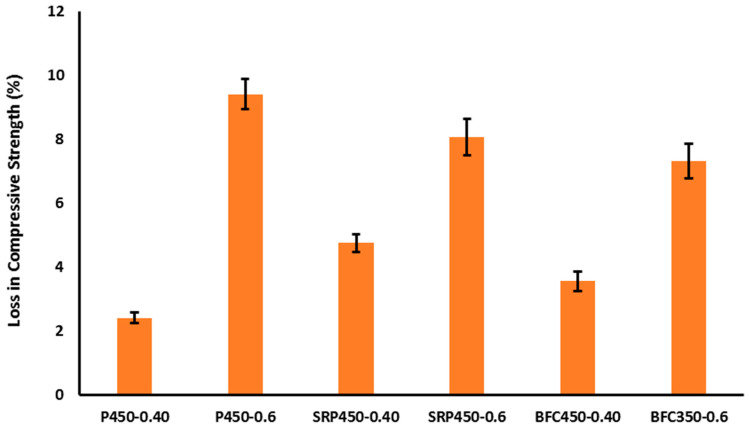
Strength loss of “high strength concrete” class after sulfate exposure.

Compressive strength results of the mixes at 28 and 180 days in water curing and 180 days in sulfate solutions for low strength concrete, medium strength concrete, and high strength concrete are given in Table 5. Compressive strength losses are showed in Figure 4, Figure 5 and Figure 6. Table 5 presents the results of compressive strength of mixes at 28 and 180 days. As observed from concrete workability, the compressive strength was directly correlated to the w/b ratio and to the strength class. For the same strength, the effect of w/b ratio on compressive strength was more pronounced with mixes that had higher strength and higher cement content. For example, the strength decreased from 19.0 to 15.4 MPa (18%) when w/b ratio increased from 0.65 to 0.8 (mixes P250-0.65 and p250-0:80). However, for mixes P450-0.4 and P450-0.6, the strength decreased from 46 MPa to 23 MPa (50%) when the w/b ratio increased from 0.4 to 0.6. As a factor that influences strength, it is undeniably the relationship between w/b ratio and porosity, since independently of all other parameters, it impacts the porosity of the cement mortar matrix and the interfacial transition region between the matrix and coarse aggregate in a concrete mix [37]. On the other hand, the influence of cement type on compressive strength is more pronounced at later ages. BFC cement exhibited the highest compressive strength compared to other types at different concrete classes. For example, it reached 64.4 MPa (BFC450-0.4), while concrete with SRP cement had 52.5 MPa and concrete with CEM I had 49.6 MPa. The strength increase with age depends on the characteristics of the used cement. Due to the high slag content in BFC cement, for mix BFC450-0.4, the 180 strength was about 124%; however, for other types of cement with fixing other parameters, the 180 strengths were 107% and 115% for CEM I and SRP cement, respectively. The slow hydration of slag reduced the strength development at early ages and improved the final strength at later ages as reflected in the experimental results. However, for the other two types of cements, the strength development is high at early ages and reduced with time.

In this investigation, the loss on compressive strength of concrete after exposure in a standardized sulfate solution was used to evaluate the performance of concrete with different types of cement under severe sulfate attack. It is clear from Figure 4, Figure 5 and Figure 6 that the strength loss due to sulfate attack was increasing with increasing w/b ratio for the same cement type. For low strength concrete, the strength loss when using different types of cement was very similar. It ranged from 5% to 6% for all cement types with w/b ratio of 0.6 and increased to 9–11% for mixes with higher w/b ratio. When increasing the w/b ratio, the microstructure of concrete became more porous and additional open channels were opened. Consequently, sulfate solution can easily penetrate the pore structure and deteriorate the compressive strength of concrete with low strength and low cement content. The role of cement type in low strength concrete was marginal due to the low content of cement associated with the high porosity and increased w/b ratio.

For medium strength concrete, the trend of strength loss was different from that of low strength concrete. The cement type as well as the w/b ratio appeared to be the governing parameters. For the same w/b ratio, the strength loss of CEM I was about twofold that of concrete made with SRP or BFC, as reflected by the results of mixes P350-0.45, SRP 350-0.45, and BFC350-0.45. When increasing the water content (w/b of 0.8), the strength deterioration of all mixes increased. For mix prepared with Portland cement, the strength deterioration reached 12.33% after sulfate exposure for 180 days. However, the resistance of sulfate attacked was better for mixes prepared with SRP and BFC since the strength loss were 7.84% and 7.16%, respectively. In the case of Portland cement, the hydration products, C-S-H, CH, and the pore structure had low tendency to resist the sulfate attack. While for concrete mixes that incorporated sulfate resistance cement or blast furnace slag cement, the hydration products included more C-S-H and less CH contents which improved the resistivity to sulfate attack. Additionally, the microstructure of SRP and BFC cements was denser and the pores were smaller, which hindered the penetration of sulfate solution significantly. Previous research asserted that large slag content in concrete enhances resistance to sulfate because the permeability of various types of water ions is decreased as a result of slag concentration [19]. The decrease in C_3_A that occurs due to a rise in the slag fraction of cement concrete is the fundamental cause of the improvement in sulfate and chloride resistance observed in cement concrete that contains a suitable amount of slag. As an added benefit, the addition of slag to concrete helps to reduce the quantities of Ca(OH)_2_ produced as a result of the reaction with slag, which leads to the creation of increased quantities of C–S–H gel that helps to increase the compressive strength [19]. According to the ACI Building Code 318-19 [33], when the sulfate level is more than 10,000 ppm in water, ASTM Type V cement with a pozzolanic admixture or slag should be used, with less than a 0.40 w/b ratio. In the current study, the trend of strength loss of high strength concrete was very close to that of medium strength concrete. The main parameters with high impact on strength deterioration after sulfate attack are w/b ratio, cement type, and concrete strength class as reflected from the obtained experimental results.

The absolute compressive strength values for different mixes are presented in Table 5. It is obvious from the table that mixes prepared with Portland cement exhibited highest deterioration and lowest strengths for the three studied concrete classes. The utilization of blast furnace slag cement showed the best strength performance. The results of compressive strength of low, medium, and high strength concrete showed the minimum deterioration after sulfate exposure for 180 days. The mix prepared with w/b of 0.4 and cement content of 450 kg/m³ (BC450-0.4) showed a compressive strength of 64.4 MPa in water curing and 62.1 MPa when cured in sulfate solution (about 3% reduction). However, when increasing the w/b ratio to 0.6, the strength loss increased to 7% (34.1 MPa and 31.6 MPa for mixes BFC450-0.6). For mixes prepared with Portland cement, the strength loss with w/b of 0.4 was 2% and increased to 9% with increasing w/b ratio to 0.6. It is notable from the obtained experimental results that, for the same type of concrete, increasing w/b ratio, increases the deterioration rate depending on the type of cement used. Incorporation of BFC cement decreased the strength deterioration of low, medium, and high strength concrete in both cases of low and high w/b ratios compared to other types of cement used.

### 4.3. Mass Change

**Figure 7:**
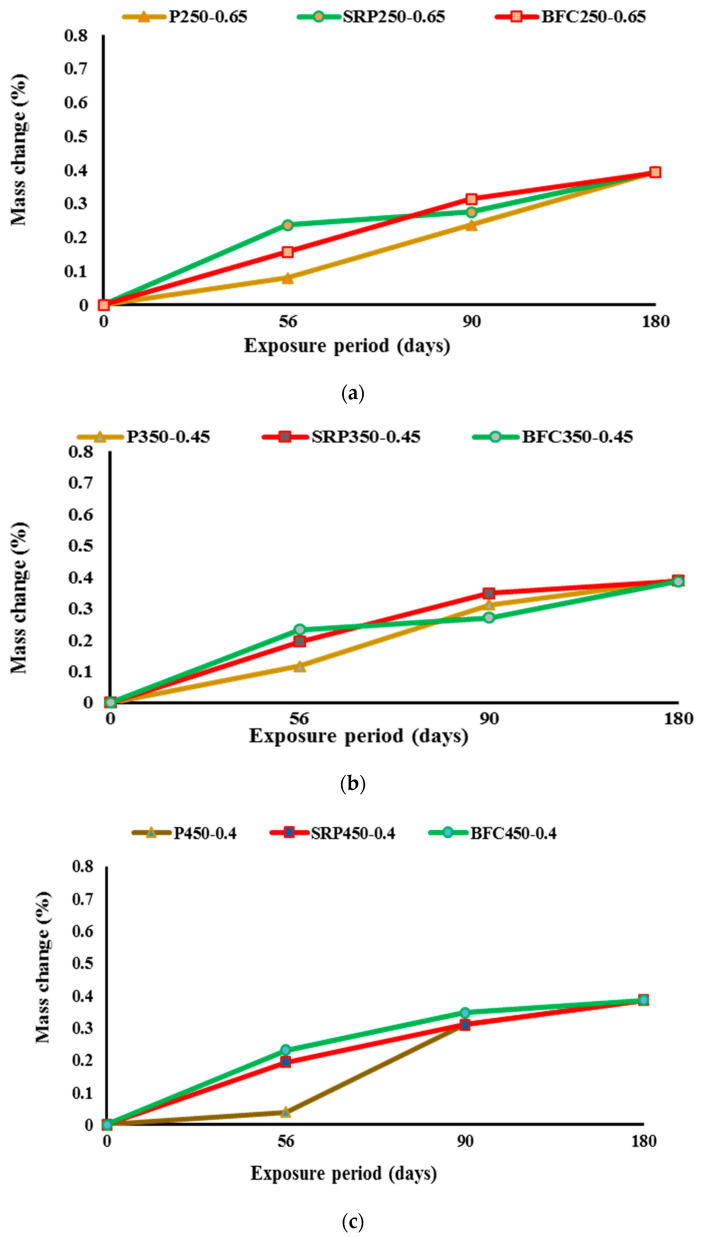
Mass changes for mixes with low w/b ratios: (**a**) low strength concrete, (**b**) medium strength concrete, and (**c**) high strength concrete.

**Figure 8:**
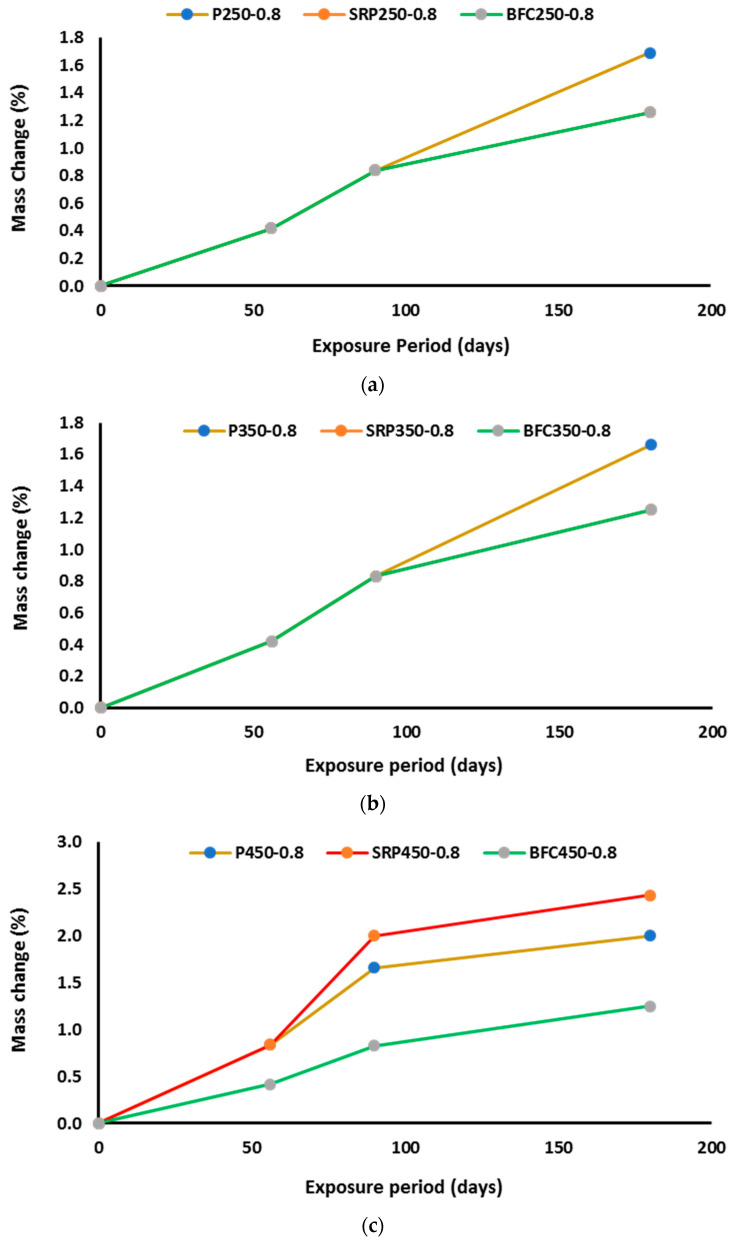
Mass change for mixes with high w/b ratios: (**a**) low strength concrete, (**b**) medium strength concrete, and (**c**) high strength concrete.

When exposed to harsh sulfate attack, concrete characteristics suffer severe deterioration with time. Mass change is considered as one of the main characteristics of concrete that can be significantly affected by exposure to sulfate solution for a long time. In this investigation, mass change with time 28, 56, 90, and 180 days were measured as can be seen in Table 6 and Figure 7 and Figure 8. For low strength concrete that had high water content (Figure 7), there were some small variations in mass change between different types of cement at early ages, while at late concrete ages (180 days), the three types of cements exhibited very close performance. When reducing the w/b ratio to 0.45, no notable influence on mass change could be detected. For medium strength concrete, the same trend was dominant. No significant change in mass change of different concretes occurred when exposing concrete to sulfate solution for a long period. For medium strength concretes, mass change depended mainly on w/b ratio and to a certain extent on the cement type. These results are in good agreement with the findings of other researchers [15]. However, the influence of cement type on mass change of concrete is obviously detectable in concrete mixes with high strength. For Portland cement concrete, the concrete mass decreased by about 2% for mix with high w/b ratio (P450-0.6), while for the same mix but with reduced w/b ratio of 0.4 (P450-0.4), the mass loss decreased to 0.4%. The use of sulfate resistance cement resulted in a similar performance to Portland cement after exposure to 180 days of sulfate solution. The mix with w/b of 0.4 (SRP450-0.4) had mass loss of about 0.4%, while with increasing w/b to 0.6 (SRP 450-0.6), the mass loss increased to 2.5%. The best performance regarding mass change was obtained from mixes prepared with blast furnace slag cement, particularly when w/b was 0.6. Mix BFC450-0.4 exhibited mass loss of about 0.4%. This can be attributed to two main reasons. The first is the low portlandite content in BFC cement due to the low clinker content in the raw materials with high replacement of slag in the production of this type of cement. The second reason could be the refinement of the pore structure associated with the use of blast furnace slag cement and with the use of low w/b ratio. When increasing w/b ratio to 0.6, the mass loss increased to about 1% which is lower than concretes prepared with Portland cement or sulfate resistance cement. It is also reported in the literature that the addition of 50% slag to standard-cured concrete improved sulfate resistance [36], which is in the same trend of findings of this investigation.

### 4.4. Scanning Electron Microscopy

**Figure 9:**
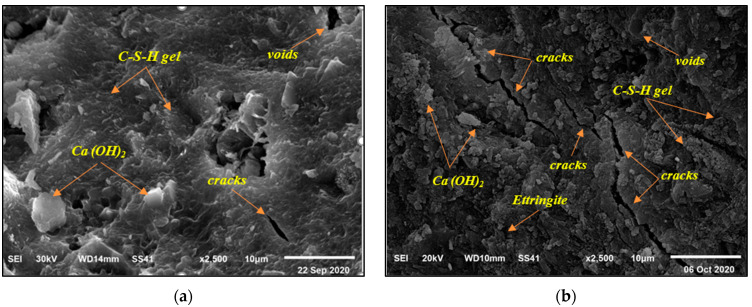
SEM images at 180 days: (**a**) mix BFC450-0.4, and (**b**) mix BFC450-0.6.

The SEM investigation unveiled diverse microstructures and morphologies of concrete specimens. This mode involves exceptional specimen preparation, such as ultra-thin sheets with extremely cleanly furbished surfaces. Nevertheless, in the instance of concrete, it was vital to observe the morphology of the surface as is, without any harm produced by specimen preparation. From the controlled mixes, mix BFC450-0.4 was selected as the testing mix. It obtained the highest compressive strength and sulfate resistance at 180 days. Figure 9 shows a dense structural microstructure for a mix of BFC and SF with HRWR at a magnification of 2500× after 180 days. On the other hand, for uncontrolled mixes, mix BFC450-0.6 achieved lower compressive strength than controlled mix BFC450-0.4. Figure 9 exhibits weakness in concrete microstructure due to sulfate attack for a mix containing BFC and SF without HRWR at a 2500× magnification at 180 days. As shown in Figure 9, more gel hydrates were seen in mix BFC450-0.4 at 180 days producing a finer and more compacted pore structure, confirming the greater compressive strength and autogenous shrinkage of the GGBS-blended cement pastes [38].

For mix BFC450-0.6 that was prepared with high w/b ratio, it can be seen that the micro-cracks were increased and spread, and the voids are shown obviously in Figure 9. When the w/b ratio was increased, the cement paste showed significantly high capillary porosity. It showed a relatively large number of important and well-connected pores. Consequently, its coefficient of permeability increased. Thus, sulfate attacks might result in the expansion and cracking of concrete. When concrete cracks, its permeability rises, and hostile water enters more easily into the inside, thereby accelerating the process of deterioration.

## 5. Conclusions

In this research, the effect of different types of cement on concrete sulfate resistance was investigated. Low strength concrete, medium strength concrete, and high strength concrete were designed, prepared, and tested. Blast furnace cement (BFC), sulfate resisting Portland cement (CEM I-SR5), and ordinary Portland cement (OPC) were used in this research. Prepared mixes were cured in water for 28 days, and then they were subjected to very severe sodium sulfate solutions (10,000 ppm) for 180 days. The performance of the fresh concrete was tested by a slump test. Compressive strength of concrete mixes was determined at 28 days. Durability was assessed using compressive strength and mass change at 180 days. Phases related to deterioration were also studied using scanning electron microscopy (SEM). The key findings and recommendations of this study are given as follows:

1. For low strength concrete, cement types showed almost similar loss in compressive strength. Therefore, any type of cement applied in this study can be used in low strength concrete to resist sulfates.
2. For medium strength concrete, mixes BFC350-0.45 and SRP350-0.45, which contained BFC and CEM I-SR5, respectively, achieved the least loss in compressive strength than that of concrete manufactured using OPC.
3. For high strength concrete, all mixes showed almost similar loss in compressive strength. Therefore, it is possible to recommend any type of cement used to resist sulfates.
4. The results confirmed that the quality of concrete, specifically with its low permeability (high cement content), is the best protection against sulfate attack.
5. The mass change due to sulfate exposure depended mainly on w/b ratio and marginally on the type of cement.
6. The SEM analysis revealed that more gel hydrates were detected in mix BFC450-0.4 that contained BFC and SF with HRWR. For high w/b mix, it could be seen that the micro-cracks were increased and spread, and the voids were shown obviously in a mix that contained BFC and SF without HRWR (mix BFC450-0.6).

## Figures and Tables

**Table 1:** Sulfate content severity (ACI-201-2008) [9].

Degree of Severity	Sulfate Solubility in Soil, % by Mass	ppm of Sulfate in Water
Mild attack	0.00–0.10	0–150
Moderate attack	0.10–0.20	150–1500
Severe attack	0.20–2.00	1500–10,000
Very severe attack	2.00–more	10,000 or even more

**Table 2:** Physical and chemical characteristics of binder used.

Types of Binder		CEM I	CEMI-SR5	BFC	SF
**Chemical characteristics**					
Lime (CaO) %		63.00	62.50	48.87	0.20
Silicon dioxide (SiO_2_)%		23.50	20.60	24.36	98.40
Aluminum oxide (Al_2_O_3_)%		6.24	4.90	3.70	0.20
Ferric oxide (Fe_2_O_3_)%		2.47	3.45	3.50	0.01
Sulfur trioxide (SO_3_)%		3.60	3.00	2.72	0.10
Tricalcium aluminate (C_3_A)%		12.60	7.15	3.80	--
**Physical and mechanical characteristics**					
Specific surface area (Blaine, m^2^/kg)		330	340	386.6	23,420
Setting time (min)	Initial	180	120	155.0	--
	Final	220	210	320.0	--
Compressive strength (MPa)	2 days	20	12	17.2	--
	28 days	48	47	46.0	--

BFC: Blast furnace cement; SF: Silica fume.

**Table 3:** Physical characteristics of aggregates used.

Physical Property	Coarse Aggregate (9.5 mm)	Coarse Aggregate (25 mm)	Normal Sand
Specific gravity	2.50	2.50	2.50
Bulk density (kg/m^3^)	1430	2200	1700
Absorption %	1.8	2.5	--

**Table 4:** Concrete mix proportions (kg/m^3^).

Mix Code	Binder Contents		Aggregate		Water	HRWR	w/b
	Cement	SF	Fine	Coarse			
P250-0.65	225	25	780	1170	163	2.5	0.65
P250-0.8	225	25	742	1112	200	--	0.80
P350-0.45	315	35	752	1128	158	3.5	0.45
P350-0.6	315	35	698	1067	210	--	0.60
P450-0.4	405	45	695	1043	180	4.5	0.40
P450-0.6	405	45	602	903	270	--	0.60
SRP250-0.65	225	25	780	1170	163	2.5	0.65
SRP250-0.8	225	25	742	1112	200	--	0.80
SRP350-0.45	315	35	752	1128	158	3.5	0.45
SRP350-0.6	315	35	698	1067	210	--	0.60
SRP450-0.4	405	45	695	1043	180	4.5	0.40
SRP450-0.6	405	45	602	903	270	--	0.60
BFC250-0.65	225	25	780	1170	163	2.5	0.65
BFC250-0.8	225	25	742	1112	200	--	0.80
BFC350-0.45	315	35	752	1128	158	3.5	0.45
BFC350-0.6	315	35	698	1067	210	--	0.60
BFC450-0.4	405	45	695	1043	180	4.5	0.40
BFC450-0.6	405	45	602	903	270	--	0.60

P: Portland cement, SRP: Sulfate resisting Portland cement, BFC: Blast furnace cement, HRWR: High-range water reducer, w/b: Water to binder ratio.

**Table 5:** Compressive strength results of all mixes in MPa.

Mix Code	Cured in Water				Exposed to Sulfate Solution	
	28 Days	SD	180 Days	SD	180 Days	SD
P250-0.65	19.0	1.3	23.6	2	22.4	1.8
P250-0.8	15.4	1.4	18.4	1.8	16.5	1.4
P350-0.45	41.7	4	47.5	1.7	42.5	2.5
P350-0.6	28.0	1.4	31.6	2.1	27.6	2.1
P450-0.4	46.0	3.1	49.6	3.3	48.4	3.1
P450-0.6	23.0	0.9	22.3	2	20.2	1
SRP250-0.65	22.7	1.8	34.0	1.5	32.0	1.5
SRP250-0.8	19.0	2.6	22.7	1.6	20.6	1.9
SRP350-0.45	45.3	3.3	51.2	3.5	48.6	3.1
SRP350-0.6	27.7	2.3	31.9	2	29.4	2.1
SRP450-0.4	45.6	4	52.5	4	50.0	3.1
SRP450-0.6	21.7	2.1	33.4	3.1	30.7	2.2
BFC250-0.65	23.7	2	35.2	3.2	33.1	2.6
BFC250-0.8	20.0	1.5	23.5	1.8	21.3	1.1
BFC350-0.45	47.7	3	54.5	2.5	51.7	2.8
BFC350-0.6	27.7	2.2	34.9	2.2	32.4	2.1
BFC450-0.4	51.7	4.8	64.4	4.3	62.1	5.3
BFC450-0.6	21.3	1.7	34.1	2.3	31.6	2.3

SD: Standard deviation in MPa.

**Table 6:** Sulfate attack influence on specimens’ weight (kg).

Mix Code	Cured in Water	Exposed to Sulfate Solution		
	28 Days	56 Days	90 Days	180 Days
P250-0.65	2.53	2.53	2.54	2.54
P250-0.8	2.37	2.36	2.35	2.33
P350-0.45	2.57	2.57	2.58	2.58
P350-0.6	2.41	2.40	2.39	2.37
P450-0.4	2.59	2.59	2.59	2.60
P450-0.6	2.45	2.43	2.41	2.40
SRP250-0.65	2.54	2.55	2.55	2.55
SRP250-0.8	2.38	2.37	2.36	2.35
SRP350-0.45	2.58	2.59	2.59	2.59
SRP350-0.6	2.42	2.41	2.4	2.39
SRP450-0.4	2.59	2.59	2.59	2.60
SRP450-0.6	2.46	2.44	2.41	2.40
BFC250-0.65	2.55	2.55	2.56	2.56
BFC250-0.8	2.39	2.38	2.37	2.36
BFC350-0.45	2.59	2.59	2.59	2.60
BFC350-0.6	2.43	2.42	2.41	2.40
BFC450-0.4	2.60	2.61	2.61	2.61
BFC450-0.6	2.47	2.46	2.45	2.44

## Data Availability

Data are contained within the article.
